# Moose and Caribou as Novel Sources of Functional Lipids: Fatty Acid Esters of Hydroxy Fatty Acids, Diglycerides and Monoacetyldiglycerides

**DOI:** 10.3390/molecules24020232

**Published:** 2019-01-10

**Authors:** Thu Huong Pham, Natalia P. Vidal, Charles F. Manful, Tiffany A. Fillier, Ryley P. Pumphrey, Karen M. Doody, Raymond H. Thomas

**Affiliations:** School of Science and the Environment/Boreal Ecosystem Research Initiative, Grenfell Campus, Memorial University of Newfoundland, 20 University Drive, Corner Brook, NL A2H 5G4, Canada; nprietovidal@grenfell.mun.ca (N.P.V.); cfmanful@grenfell.mun.ca (C.F.M.); tfillier@grenfell.mun.ca (T.A.F.); rpp877@mun.ca (R.P.P.); kdoody@grenfell.mun.ca (K.M.D.)

**Keywords:** functional lipids, functional foods, fatty acid esters of hydroxy fatty acids, diglycerides, regioisomers, monoacetyldiglycerides

## Abstract

Fatty acid esters of hydroxy fatty acids (FAHFA), diglycerides (DG) and monoacetyldiglycerides (MAcDG) are gaining interest as functional lipids in pharmaceuticals and functional food formulations for managing and treating metabolic or inflammatory diseases. Herein, we investigated whether the antler and/or meat of two Cervids (moose and caribou) are novel sources of FAHFA, DG and MAcDG. We observed FAHFA present in moose and caribou composed mainly of polyunsaturated families, and that the esterification occurred frequently at the C5-hydroxy fatty acid moiety, most noticeably arachidonic acid 5-hydroxyeicosatrienoic acid (ARA-5-HERA). Moose antler, caribou and moose meat also contained significant levels of both 1,2-DG and 1,3-DG lipids. The 1,3-DG molecular species consisted mainly of 16:0/18:1, 18:0/16:0, and 18:0/18:1. On the other hand, major 1,2-DG species consisted of DG 18:0/18:0, 16:0/16:0 and 18:1/18:1 molecular species with higher levels in the antler compared to the meat. The molecular species composition of MAcDG was very simple and consisted of 14:2/18:2/2:0, 16:0/18:2/2:0, 16:0/18:1/2:0 and 18:0/18:1/2:0 with the first species 14:2/18:2/2:0 predominating in the tip of moose antlers. Increasing access to and knowledge of the presence of these functional lipids in foods will enhance their intake in the diet with potential implications in improving personal and population health.

## 1. Introduction

The increasing recognition of the role of dietary fatty acids in enhancing personal and population health by reducing the risk factors for many common illnesses has led to consumers demanding meat products with improved fatty acid composition [1,2,3]. Big game Cervids such as moose (*Alces alces*) and caribou (*Rangifer tarandus*) are gaining popularity as excellent sources of low-fat lean meat containing superior fatty acid profiles (balanced omega 6:3 essential fatty acids) compared to traditional farm raised or domesticated meat animals [4].

The superior fatty acid profiles of wild big game Cervids are attributed to the forage they consume as a normal part of their diet [5]. This also lends the possibility that due to the diverse kinds of forages consumed by Cervids, they may be excellent or novel sources of low abundance and/or uncommon modified lipids such as fatty acid esters of hydroxy fatty acid (FAHFA), diglycerides (DG) or monoacetyldiglycerides (MAcDG). Based on the modifications in the fatty acid structures of these low abundance or uncommon lipids they have unique or value-added properties, which have been demonstrated to confer potential health benefits in humans [6,7,8]. As such, there are several applications and uses of these lipids as the bioactive ingredients in functional foods or pharmaceuticals [7,8,9,10,11,12].

MAcDG was first identified in Japanese deer (*Cervis nippon*) antlers [13]. To date, cow udder [14] and the larvae of the golden rod fly (*Eurosta solidaginis*) [15] represent the only two other animal sources of MAcDG reported in the literature. MAcDG is a unique form of triglyceride containing acetate at stereospecifically numbered carbon 3 (*sn-3*) of the glycerol moiety instead of longer chain fatty acids (C4–24) typically found in common triglycerides [10,15,16]. The presence of acetate at *sn-3* of the glycerol backbone confer unique chemical properties and uses to this compound. For example, emerging evidence suggest roles in promoting or suppressing immune functions, inhibition of tumor growth, treatment of sepsis and cancer [10,11,17]. Recently, MAcDG was used as the active ingredient in pharmaceuticals and functional food composition for preventing and/or treating rheumatoid arthritis [6]. In this invention, MAcDG was reported to be effective in inhibiting the phosphorylation of STAT-3, the known therapeutic target for rhematoid arthritis [6]. Furthermore, MAcDG has been demonstrated to be effective in treating sepsis [17], inflammation [10] and asthma [11].

Like MAcDG, diglycerides (DG) are presented as low abundance lipids in food products, but have significant potential or roles in health maintenance, as well as reducing the risk factors for several chronic diseases [7]. In particular, DG has been shown to improve post prandial hyperlipidemia, which has been identified as a risk marker for cardiovascular diseases [7]. DGs are structurally distinct from triglycerides (TGs) in that only two fatty acids are esterified at either *sn-1/2* or *sn-1/3* of the glycerol moiety in DG compared to three fatty acids esterified into TGs. This esterification leads to the formation of 1,2 and 1,3-DG molecular species, which have metabolic and nutritional characteristics distinct from that of triglycerides [18,19]. For example, several nutritional and clinical studies have demonstrated that DGs (particularly the 1,3-DG) are less likely to be stored as body fat compared to triglycerides [7,18,19]. As such, DG has been shown in several clinical trials to reduce body weight accumulation following the consumption of DG enriched oil-based diets [7,20,21]. The ability of DG to suppress both obesity and post prandial hyperlipidemia, known risk factors for developing diabetes and cardiovascular diseases respectively, have resulted in the incorporation of DG in many food products [22,23]. Currently, DG content is enhanced as a functional ingredient in many edible oils (functional oils) and functional food products to capitalize on these health benefits [19,24].

FAHFAs consist of a fatty acid esterified to a hydroxy fatty acid (HFA) and was first identified in 2014 as a novel class of lipids in mice enhancing insulin secretion and glucose tolerance [8,9]. The esterification can occur to the hydroxy substituent at different positions, e.g., C5, C7, C8, C9, C10, C12 or C13 on the hydroxy fatty acid moiety forming several families of FAHFAs. For example, if palmitic acid is esterified to hydroxy stearic acid, the resultant family of FAHFA is denoted as palmitoyl hydroxy palmitoleic acid (PAHSA). Saturated hydroxy fatty acids tend to predominate the families of FAHFAs reported in the literature with PAHSA and OAHSA (oleoyl hydroxy stearic acids) being the two most abundant families [9,25]. Of these, the saturated families tend to predominate with 9- and 5-palmitic acid ester of hydroxy stearic acids (9- and 5-PAHSA regioisomers) showing significant potential for treating type 2 diabetes [8,25,26]. In addition, unsaturated FAHFAs have been demonstrated in patients, cells and animal models to be very effective in reducing or impeding inflammation associated with ulcerative colitis and chronic low-grade inflammation in obese patients with type 2 diabetes which suggest potential applications of FAHFAs. For example, these FAHFAs were reported to suppress the number of macrophages positive for tumor necrosis factor (TFNα) and interleukin 6 (IL-6); prevented the activation of the immunosuppressive enzyme indole amine 2,3-dioxygen in human blood; and decreased T-cell activation and colitis associated inflammation in a mouse model of ulcerative colitis [9,25]. Furthermore, polyunsaturated (PUFA) families of FAHFAs, such as 13-docosahexaenoic acid hydroxylinoleic acid (13-DHAHLA), were demonstrated to be very effective in suppressing inflammation. These findings suggest the bioactivities of FAHFAs could also have potential therapeutic applications in the treatment of inflammatory diseases. It appears reduced endogenous levels of FAHFA’s, particularly PAHSA, could contribute to increased risks of developing diabetes and inflammatory diseases [25,27].

Taking these into consideration, FAHFAs, DGs and MAcDGs, are gaining interest as functional lipids in pharmaceuticals and functional foods formulations for managing and treating metabolic or inflammatory diseases including obesity, type 2 diabetes, sepsis and rheumatoid arthritis [6,9]. There is also huge interest in the scientific community to assess different foods as novel sources of these functional lipids for potential as natural ingredients in functional food formulation or to determine new uses for these foods in the niche functional foods market. The observation that DG and FAHFAs are present in some foods albeit in low concentrations [7,8,9], and that the antlers of red deer (Cervid) is the first of two mammalian sources of MAcDG previously reported in the literature [13,15], we hypothesize that Cervids could be novel sources of FAHFAs, DG and MAcDG based on their diet and genetic relationship to red deer. Herein, we investigated whether the antler and/or meat of two Cervids (moose and caribou) are novel sources of FAHFAs, DG and MAcDG. To the best of our knowledge, this is the first study reporting the presence of these functional lipids in moose and caribou meat or antler.

## 2. Experimental Section

### 2.1. Meat and Antler Acquisition

Moose and caribou meat or antlers were obtained from the Newfoundland and Labrador Department of Natural Resources. Hunters were asked to donate 2–4 lbs. of meat, as well as antlers from each harvest. Ethics approval for this study was granted by Memorial University Animal Care Committee as mandated by the Canadian Council on Animal Care and all the experiments were performed in accordance with relevant guidelines and regulations. Animals were harvested at different locations across the Province and the samples (meat and antler) taken at butcher shops when the animals went in for processing. Samples were labeled as follows: Location/date of harvest, sex, and meat cut. Samples were wrapped in brown paper, frozen and delivered to the lab for lipid analysis. The hunters were only able to provide antlers from moose. As such, no caribou antler was evaluated in this study. Similarly, both male and female meat samples were accessible only for moose. Consequently, we were only able to evaluate both sexes in moose.

### 2.2. Lipid Analysis

Frozen moose antlers were cut with a band saw and the powder obtained from sawing was weighed (100 mg) and directly used for lipid extractions. The base (MA-B) and tip (MA-T) of antlers from four animals were used for the extractions. Similarly, meat (100 mg) obtained from four male (MM-M) or female moose (MM-F) and male caribou (CM) were cryo-homogenized and extracted prior to lipid analysis. The samples were extracted using a modified Bligh and Dyer method (Bligh & Dyer, 1959). Briefly, 1 mL methanol containing 0.01% butylated hydroxytoluence (BHT) is addeda, followed by 1 mL chloroform and 0.8 mL water, homogenized using an Omni tip probe (Omni Tissue Homogenizer, Fisher Scientific, ON, Canada). The mixture was then centrifuged for 10 min at 5000 rpm. The organic phase (bottom layer of the centrifuge tube) consisting of total lipids was collected and transferred to pre-weighed glass vials and dried under nitrogen gas. Lipids extracted were re-suspended in methanol:chloroform (1:1 *v*/*v*) to a desired concentration.

#### Mass Spectrometry

**Figure 1:**
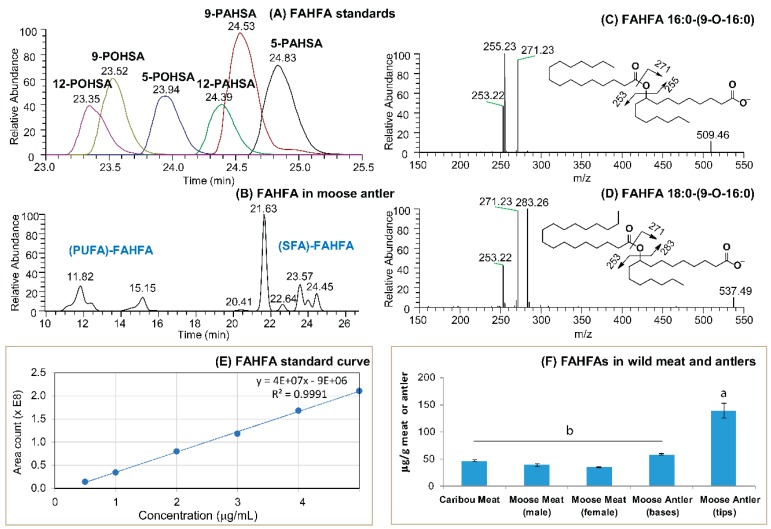
Confirmation of the presence of fatty acid ester of hydroxy fatty acids (FAHFAs) in moose or caribou meat and moose antler. UHPLC-C30RP-MS chromatographic separation of (**A**) FAHFAs commercial standards and (**B**) FAHFAs in a moose antler sample; (**C**,**D**) LC-MS/MS mass spectra represents mass spectrum showing the fragmentation patterns of two major FAHFA species present in moose meat or antler samples; (**E**) external standard curve used to calculate the amount of FAHFAs present in caribou and moose meat or antler samples; (**F**) the total amount of FAHFAs present in moose or caribou meat or moose antler samples. Values (µg/g meat or antler) represent means ± SE, n = 8 samples per treatment. Means are significantly different at α = 0.05 and are denoted by different letters (a, b). (PUFA)-FAHFA = Polyunsaturated fatty acid based FAHFA, (SFA)-FAHFA = Saturated fatty acid based FAHFA, POHSA = palmitoleyl hydroxy stearic acid, PAHSA = palmitoyl hydroxy stearic acid, number included before the abbreviation, e.g., 9-PAHSA or PA-9-HSA denotes that palmitic acid esterified at the 9-position of hydroxy stearic acid counting from the carboxylate functional group.

Samples was analyzed according to the detailed methods in (Narvaez-Rivas & Zhang, 2016) [28] with the following modifications: The extracted lipids were resolved on an Accucore C30 reverse phase column (150 × 2 mm I.D., particle size: 2.6 µm, pore diameter: 150 Å; ThermoFisher Scientific, ON, Canada) coupled to a Dionex Ultimate 3000 ultra-high performance liquid chromatography (UHPLC) system and a Q-Exactive Orbitrap high resolution mass spectrometer (ThermoFisher Scientific, Toronto, ON, Canada). The solvent systems used for separation was as follows: Solvent A consisted of acetonitrile:water (60:40 *v*/*v*), while solvent B consisted of isopropanol:acetonitrile:water (90:10:1 *v*/*v*/*v*); with both solvents containing 10 mM ammonium formate and 0.1% formic acid. The gradient used for separation was as follow: 30% B for 3 min; 43% B for 5 min, 50% B for 1 min, 90% B for 9 min, 99% B for 8 min, 99% B for 4 min and re-equilibrated at 70% A for 5 min. The column temperature was set at 30 °C with a flow rate of 0.2 mL/min, and 10 µL of the sample or standards injected in the instrument. The mass spectrometer was operated in both positive and negative ion modes using the following parameters: sheath gas: 40, auxiliary gas: 2, ion spray voltage: 3.5 kV, capillary temperature: 300 °C; S-lens RF: 35 V; mass range: 200–2000 *m*/*z*; full scan mode at a resolution of 70,000 *m*/*z*; top-20 data dependent MS/MS at a resolution of 35,000 *m*/*z* and step collision energy of 35 and 40 (arbitrary unit); injection time 50 min; isolation window: 1 *m*/*z*; automatic gain control target: 1e5 with dynamic exclusion setting of 5.0 s. The Instrument was externally calibrated to 1 ppm using ESI negative and positive calibration solutions (ThermoScientific, Waltham, MA, USA). In addition, confirmation of the FAHFA positional isomers (Figure 1A) were determined following direct infusion and MS/MS experiments.

### 2.3. Lipid Standards

FAHFA standards (12-POHSA, 9-POHSA, 5-POHSA, 12-PAHSA, 9-PAHSA and 5-PAHSA), and DG standards (1,2-DG 18:0/20:4 and 1,3-18:1/18:1) were purchased from Avanti Polar Lipids, Alabama, USA. Purified MAcDG standards (16:0/18:1/2:0) was provided by Chemforce Laboratories, Edmonton, AL, Canada. The standards were used to generate standard curves for quantification of the FAHFAs, DG and MAcDG in the samples.

## 3. Results and Discussions

Cervids refer to animals of the *Cervidae* family and include species such as deer, elk, moose, and caribou. These species are popular big game species present throughout the circumpolar boreal forests of Eurasia and North America, such as Canada, Alaska, New England [29]. Though these species are typically wild and free ranging, in many countries they are farmed, and the meat used as high-quality protein sources [4], and the antlers used for medicinal purposes [12]. In fact, Cervid farming is a big business in the USA with over 7000 farm operations and 2000 hunting reserves reported across the country [30]. Moreover, the current global trend indicates these operations, as well as animals harvested from the wild are increasing consistent with the growing demand and consumption of the meat from these animals as a high quality protein source [31]. Furthermore, the demand for healthy food choices by a more health conscious consumer base with increasing knowledge of the association between the consumption of functional foods and reduced disease risks also contributes to the rising demand for wild or farmed Cervid meat as a high-quality meat source. In fact, wild game meat from Cervids such as moose and caribou are highly valued by consumers because of its unique taste, low fat content, balanced ratio of omega 6 to 3 essential fatty acids, high protein content and perceived positive effects on cardiovascular health [4,31]. Additionally, deer velvet antlers are widely used in many cultures as sources of traditional or folk medicine [10,17]. MAcDG was isolated from deer velvet antler as the active ingredient responsible for the positive health benefits associated with deer velvet antler use in traditional Korean medicine [6,10]. This led us to investigate whether moose and caribou, close relatives of deer also contained MAcDG, as well as the recently discovered FAHFA along with DG.

### 3.1. FAHFAs as Functional Lipids in Moose and Caribou

FAHFA is an important class of functional lipids demonstrated to stimulate insulin secretion, improve glucose tolerance and insulin sensitivity in diabetic patients and animal models of diabetes [8,9,25]. The presence of FAHFA was detected in both caribou and moose meat, as well as in moose antlers (Figure 1). Separation of FAHFA standards is demonstrated in Figure 1A based on the position of the hydroxy group on the hydroxy fatty acid backbone and degree of unsaturation. The extracted ion chromatography of FAHFAs in a moose antler sample is shown in Figure 1B and MS/MS spectra in Figure 1C,D represent the presence and fragmentation patterns of two major FAHFA species in moose meat or antler samples. The external standard curve (Figure 1E) was used to calculate the amount of FAHFAs.

**Figure 2:**
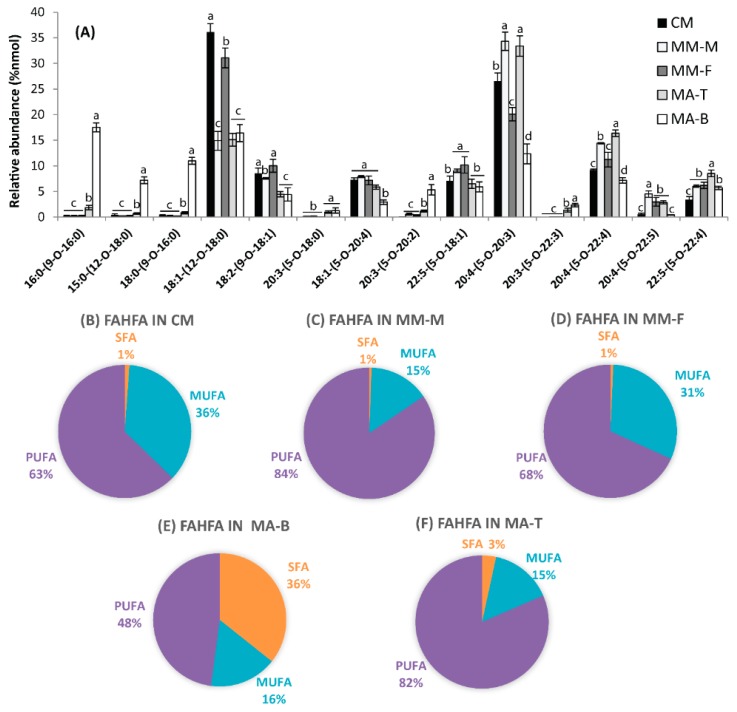
Molecular species composition and relative abundance of FAHFAs in moose and caribou meat or moose antler samples. Values represent means ± SE, n = 8 samples per treatment. Means are significantly different at α = 0.05 and are denoted by different letters (a–d). (**A**) Molecular species composition of FAHFAs present in moose and caribou meat and moose antler samples. (**B**–**F**) shows proportion of saturated, munounsaturate and polyunsaturated FAHFAs in moose and carbou meat and moose antlers. CM = Caribou Meat, MM-M = Moose Meat (male), MM-F = Moose meat (female), MA-T = Moose Antler (tips), MA-B = Moose Antler (bases), SFA = saturated fatty acids, MUFA = monounsaturated fatty acids, PUFA = polyunsaturated fatty acids.

Both caribou and moose meat had similar levels (Figure 1F) and molecular species compositions of FAHFA (Figure 2). However, moose antler contained significantly higher levels of total FAHFAs compared to the meat of both animals (Figure 1F). The tip of the antler had the highest level, and it was four-fold higher compared to either moose or caribou meat. The FAHFA level in the base of the antler was lower than in the tip, but higher than the levels in the meat. Interestingly, a greater proportion of saturated FAHFAs was detected in the base of the antler compared to the tip or the meat (Figure 2) with 9-PAHSA (16-0-(9-O-16:0) predominating. The position of hydroxy groups on HFA backbone was identified based on established rules [32]. From the best of our knowledge, this is the first report documenting the presence of FAHFA in Cervids. These findings are consistent with previous reports in the literature where FAHFAs were found in foods including apple, broccoli, beef, chicken and eggs [8,9,32]. The quantity of these compounds observed in the evaluated food samples were at similar levels (pmol/g) to what we report in our study (µg/g) for moose antler, caribou and moose meat.

The molecular species composition of FAHFA in moose and caribou were characterized by UHPLC-MS/MS and the diagnostic fragmentation used for characterization summarized in Table 1. FAHFAs present in moose and caribou were composed mainly of polyunsaturated families, and the esterification occurred predominantly at the C5-hydroxy substituent on the HFA moiety (Table 1, Figure 2A). Interestingly, the polyunsaturated FAHFAs accounted for 48% of the total FAHFAs present at the base of the antler (MA-B), compared to 82% at the tip (MA-T), 84% in male moose meat (MM-M), 68% in female moose meat (MM-F), and 63% in caribou meat (CM); with arachidonoyl 5-hydroxyeicosatrienoic acid (ARA-5-HERA) predominating (Figure 2B–F). Polyunsaturated families of FAHFAs have been demonstrated to be very effective in suppressing inflammation [8,9,25,27]. Knowledge of the composition and levels of FAHFAs in natural food sources could be beneficial in making informed choices associated with including superior food sources of FAHFAs in the daily diet.

Collectively, this work demonstrates for the first time that moose and caribou meat and moose antlers are natural source of FAHFAs enriched with polyunsaturated fatty acids. These findings may have potential significance in functional food applications or nutraceutical formulations considering emerging evidence demonstrating the beneficial effects of oral administration of FAHFAs in stimulating glucose tolerance and insulin secretion in patients and animal models of diabetes or inflammation.

### 3.2. DG as Functional Lipid in Moose and Caribou

**Figure 3:**
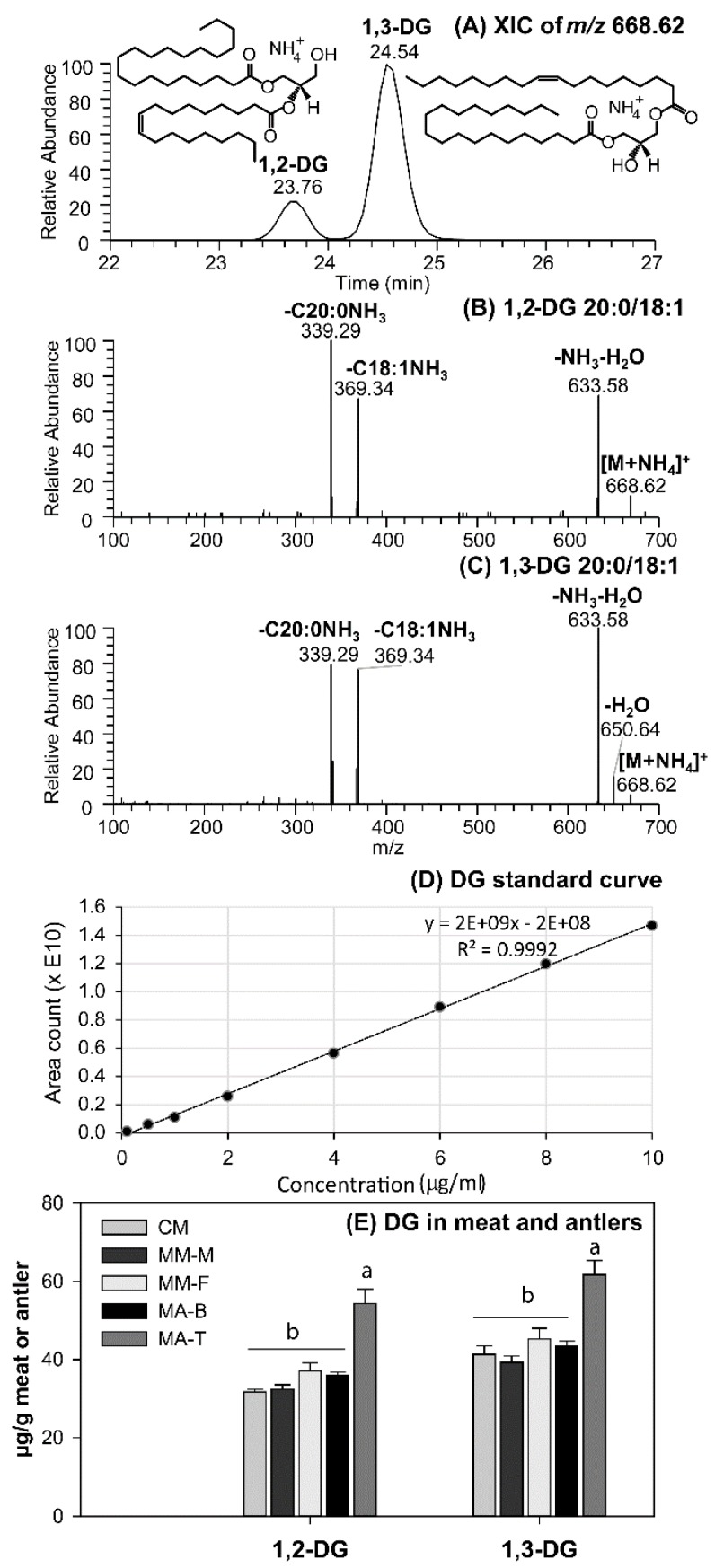
Separation and detection of 1,2- and 1,3-diglycerides (DGs) in moose or caribou meat and moose antler by liquid chromatography and mass spectrometry. (**A**) UHPLC-C30RP-MS extracted ion chromatographic (XIC) separation of the 1,2- and 1,3-DG (20:0/18:1) isomers [M + NH_4_]^+^ *m*/*z* 668.62; (**B**,**C**) MS/MS mass spectra showing the fragmentation patterns of 1,2- and 1,3-DG (20:0/18:1) isomers found in wild meat; (**D**) standard curve used to calculate the amount of DG present in caribou and moose meat or antler samples; (**E**) the total amounts of 1,2 and 1,3-DGs present in moose or caribou meat or moose antler samples. Means are significantly different at α = 0.05 and are denoted by different letters (a, b). CM = Caribou Meat, MM-M = Moose Meat (male), MM-F = Moose meat (female), MA-T = Moose Antler (tips), MA-B = Moose Antler (bases).

Diglycerides is a class of functional lipids and are more commonly found in foods compared to FAHFAs and MAcDGs. Although DGs typically exist as minor components in natural food sources, they have demonstrated significant health benefits [7,18,19]. Moose antler, caribou and moose meat were found to contain significant levels of both 1,2- and 1,3-DG molecular species (Figure 3).

**Figure 4:**
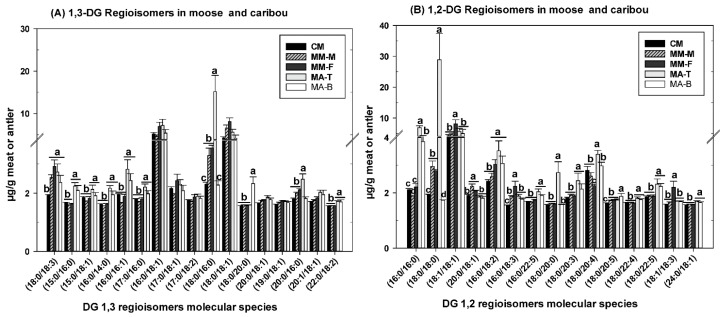
Molecular species composition and relative abundance of (**A**) 1,3-DGs and (**B**) 1,2-DGs in moose and caribou meat or moose antler samples. Values represent means ± SE, n = 8 samples per treatment. Means which are significantly different (α = 0.05) are denoted by different letters (a–c). 1,2-DG represents diglycerides with the fatty acids esterified at *sn-1/2* of the glycerol moiety. 1,3-DG represents diglycerides with the fatty acids esterified at *sn-1/3* of the glycerol moiety. CM = Caribou Meat, MM-M = Moose Meat (male), MM-F = Moose meat (female), MA-B = Moose Antler (bases), MA-T = Moose Antler (tips).

The 1,2-DG molecular species containing 2 different fatty acyl chains could be delineated from the 1,3-DG species based on the proportion of the fragment ions representing the neutral fatty acid losses. Separation of the 1,2-DG and 1,3-DG isomers were achieved by C30 reverse phase liquid chromatography (C30RPLC—see Experimental Section) as an example shown in Figure 3A, where the 1,2-DG (20:0/18:1) species was eluted before their 1,3-partner, further assisting the identification of the DG molecular species. In MS/MS spectra of the 1,2-DG molecular species, the peak representing the loss of fatty acids at *sn-2* is generally lower than that of the *sn-1* fatty acid loss (Figure 3B) while the 1,3-DG species produces fragment ions representing the *sn-1* and *sn-3* fatty acid losses with similar intensities (Figure 3C) [33,34,35]. Consistent with previous reports in the literature [7,19], the abundance of 1,3-DG were higher than the 1,2-DG in all the samples evaluated (Figure 3D,E). The moose antler tips (MA-T) contained the most DG (including both types) followed by the base of the antler. However, the level of DG in the base of the antler was similar to that of both caribou and moose meat. The meat of moose and caribou also contained similar levels of DG inclusive of both 1,2- and 1,3-DG molecular species. This raises the possibility that DG obtained from moose or caribou meat fat and moose antlers could have potential applications in the development of cervid meats or antlers based functional foods. In this study, the fatty acid composition varied dramatically between the 1,2 and 1,3-DG species in both meat and antlers. The 1,3-DG molecular species consisted predominantly of DG 16:0/18:1, 18:0/16:0, and 18:0/18:1 molecular species with higher levels generally in the tip of the antler compared to the meat or the base (Figure 4A). On the other hand, the 1,2-DG molecular species consisted mainly of DG 16:0/16:0, 18:0/18:0, and 18:1/18:1 molecular species with higher levels in the antler compared to the meat (Figure 4B).

Though there was distinct variation in the fatty composition between the 1,2- and 1,3-DG molecular species and their levels in the antler and meat, it is the specific structural differences of the DG lipids rather than the fatty acid composition that confer the beneficial effects on lipid metabolism and body weight [21]. Consumption of DG in the diet is generally very low because of the inherently low concentration present in most natural food sources. Knowledge of the DG content in different food sources present an opportunity to better incorporate DG in the diet. It also presents the opportunity to developed new foods or enhanced the content of DG as functional ingredients in existing food products. Increased access to food products enriched with DG could aid in increasing the intake of DG in the diet; with potential implications in managing post prandial lipidemia, and obesity identified as high-risk factors in developing cardiovascular disease and type 2 diabetes [7].

### 3.3. MAcDG as Functional Lipids in Moose and Caribou

**Figure 5:**
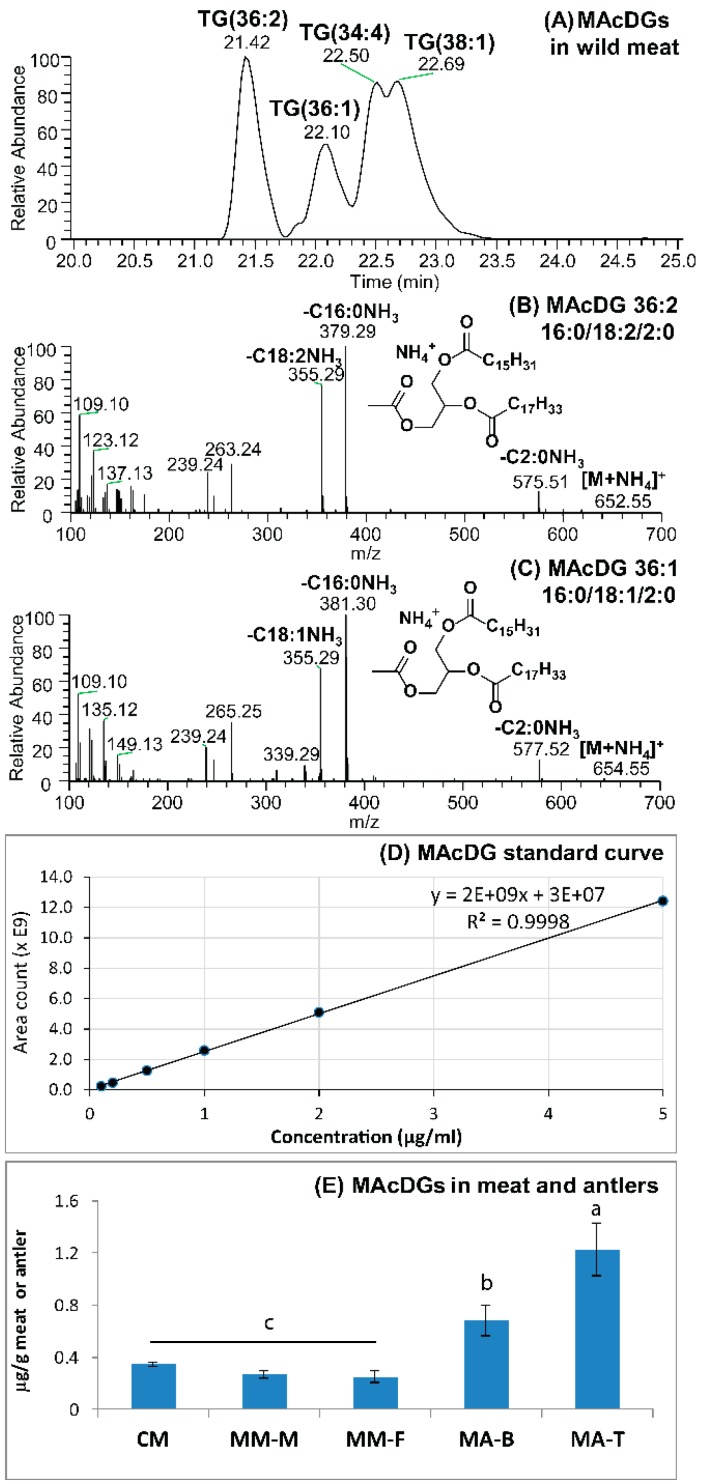
Separation and detection of monoacetyldiglycerides (MAcDG) in moose or caribou meat and moose antler. (**A**) UHPLC-C30RP-MS chromatographic separation of MAcDGs in a moose meat sample; (**B**,**C**) MS/MS mass spectra showing the fragmentation patterns of two major MAcDG molecular species; (**D**) external standard curve used to calculate the total amounts of MAcDG present in caribou and moose meat or antler samples; (**E**) the total amounts of MAcDG present in the samples. Means are significantly different at α = 0.05 and are denoted by different letters (a–c). CM = Caribou Meat, MM-M = Moose Meat (male), MM-F = Moose meat (female), MA-B = Moose Antler (bases), MA-T = Moose Antler (tips).

One of the most striking findings in this study is the presence of MAcDG in both moose and caribou meat, as well as, moose antler. This is the first time to our knowledge that MAcDG has been reported in meat in any animals (Figure 5A–D). We observed that MAcDG was present in moose and caribou and moose meat, albeit in a low concentration (Figure 5E). It is important to note even though the level is low, they exist within the range of signaling lipids that can possess very potent bioactivities at these low concentrations.

**Figure 6:**
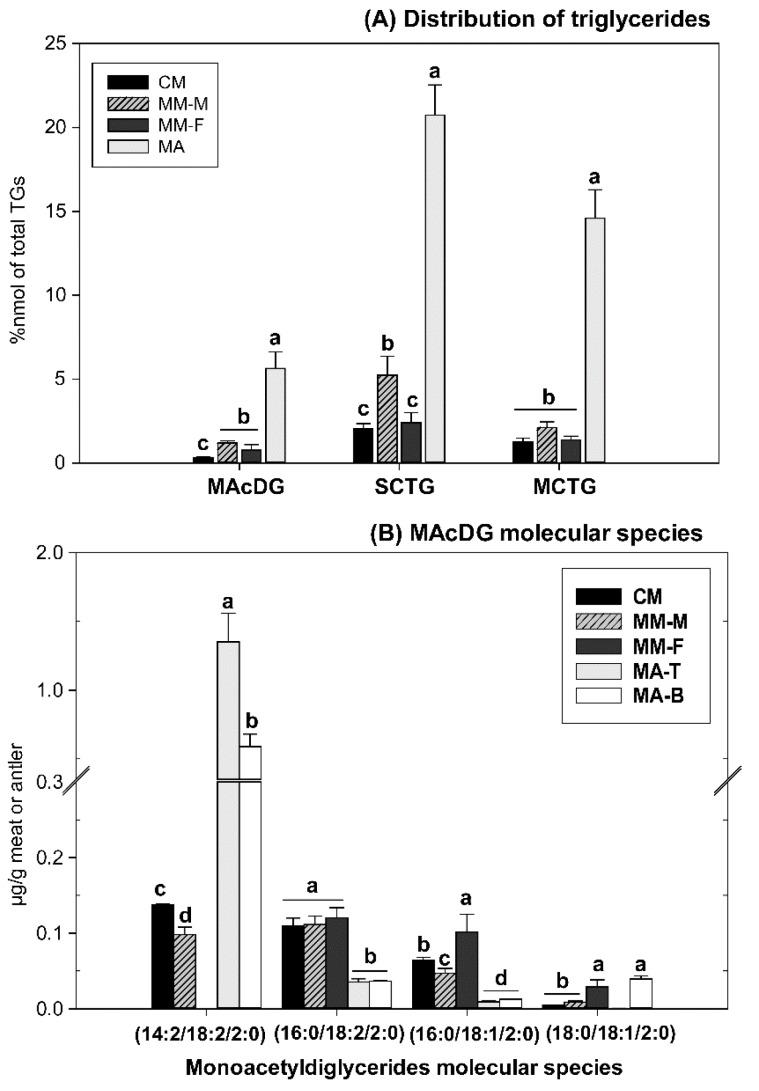
(**A**) Relative quantitation of MAcDG, SCTG and MCTG of total triglycerides and (**B**) Molecular species composition and relative abundance of monoacetyldiglycerides (MAcDGs) in moose and caribou meats or moose antler samples. Values represents nanomole percent of the MAcDG. Values represent means ± SE, n = 8 samples per treatment. Means are significantly different at α = 0.05 and are denoted by different letters (a–d). TG = triglyceride or commonly known as triacylglycerol, MAcDG = monoacetyldiglyceride, SCTG = short-chain triglyceride, MCTG = medium-chain triglyceride. CM = Caribou Meat, MM-M = Moose Meat (male), MM-F = Moose meat (female), MA = Moose Antler, MA-B = Moose Antler (bases), MA-T = Moose Antler (tips).

The tip of the antler contained the highest level followed by the base; with the lowest level recorded in moose meat (Figure 5E). The level in caribou meat was higher than that in moose meat. Interestingly, deer antler is renowned in traditional oriental medicine in Korea for having numerous medicinal benefits including improving cardiac functions, relieving fatigue, enhance immunity and treating nervous breakdown [10]. Many studies have been conducted to determine what are the active ingredient(s) contributing to the beneficial effects associated with the use of deer antler in traditional oriental medicine in Korea. This has led to the discovery of MAcDG in deer antlers and demonstration of its potent bioactivities against inflammation related illnesses; and subsequently it was used in developing health or functional foods and pharmaceutical compositions specifically for treating and managing these illnesses [6,10,11,17]. This study confirms for the first time the existence of MAcDG in moose antler, as well as, in caribou and moose meat (Figure 5 and Figure 6). This finding opens the possibility and presents opportunities for the potential use of MAcDG from moose and caribou in functional food development. There is also very little information about MAcDG in the literature as a functional ingredient, as well as, potential health benefits. The hope is this work will stimulate more awareness and investigations across the scientific community regarding the use of MAcDG in human health therapy.

MAcDG is a triglyceride with the presence of acetate at *sn-3* of the glycerol moiety compared to 3 or more carbon in short (3–5 carbons), medium (6–12 carbons) and long chain triglycerides (13–26 carbons) [6,10,15,16]. As such, we characterized the distribution of MAcDG in moose antler, moose and caribou meat in relation to medium and short chain triglycerides present in the total triglycerides (Figure 6A). Approximately, 1% of the total triglyceride composition in meat was detected as MAcDG, while it accounted for about 4% in moose antlers (Figure 6A). The literature is replete with the health benefits associated with the bioactivities and consumption of medium chain triglycerides [36]. The amount of medium chain triglycerides (MCTG) in moose antlers was approximately 15% of the total triglycerides, while the short chain triglycerides (SCTG) contributed about 20%.

These findings further support potential applications for the use of moose antlers as natural sources of functional lipids in functional foods formulation or development. The molecular species composition of MAcDG was characterized by MS/MS with their diagnostic ions listed in Table 2. The MAcDG profile was very simple and consisted of 18:2/14:2/2:0, 18:2/16:0/2:0, 18:1/16:0/2:0 and 18:1/18:0/2:0 with 18:2/14:2/2:0 predominating in moose antler (Figure 6B). Meat had significantly lower levels of 18:2/14:2/2:0 compared to the antler. Conversely, 18:2/16:0/2:0, 18:1/16:0/2:0 and 18:1/18:0/2:0 were generally higher in the meat compared to the antler (Figure 6B). Taken all together, these findings demonstrate the occurrence of MAcDG in moose and caribou with significantly higher levels in the antlers compared to the meat. The unique composition and biochemical properties of MAcDG confer novel uses or applications in the healthcare field (modulate immune functions, inhibit tumor growth, potential treatment of sepsis and cancers) [6,10,11,17], and biofuel industry (possible additive to enhance efficiency of biofuels when used in cold climate applications) [15,16]. These uses suggest possible applications for MAcDG from moose and caribou moose in these industries.

## 4. Conclusions

This work shows for the first time the existence of FAHFAs, MAcDG and DG in moose antlers, caribou and moose meat. Generally, the antler had higher levels of these lipids compared to the meat. However, qualitatively, the same molecular species were present in both antler and meat. FAHFAs, MAcDG and DG are new classes of functional lipids demonstrated to have potential therapeutic significance in the management and prevention of metabolic or inflammatory diseases including obesity, type 2 diabetes, sepsis and rheumatoid arthritis. These findings support potential applications for the use of moose antlers, moose and caribou meat containing MAcDG, FAHFA and DG as natural sources of functional lipids in functional food formulations or development. Collectively, this work demonstrates for the first time that moose and caribou meat and moose antlers are natural sources of FAHFAs enriched with polyunsaturated fatty acids. These findings may have potential significance in functional food applications or nutraceutical formulations considering the emerging evidence demonstrating the beneficial effects of oral administration of FAHFAs in stimulating glucose tolerance and insulin secretion in patience and animal models of diabetes or inflammation [8,9,25,27]. High level (µg/g) quantities of DG, particularly, the 1,3-DG molecular species were observed in both moose antler, caribou and moose meat. The 1,3-DG lipids are reported to be the most effective in reducing post prandial serum triacylglycerol level and suppress obesity, known risk factors for developing diabetes and cardiovascular diseases. In this study we show for the first time the presence of MAcDG in mammalian meat (moose and caribou), as well as in moose antler. Although this work is descriptive in nature, it fills a gap related to the lack of information present in the scientific literature regarding the extent to which low abundance modified or uncommon lipids existing in difference food sources. Emerging evidences suggest that these lipids have significant beneficial effects at low concentrations in reducing the risk factors for common lifestyle related illness such as diabetes, obesity, and cardiovascular diseases. Increasing access to and knowledge of the presence of these functional lipids in foods will enhance their intake in the diet with potential implications in improving personal and population health.

## Figures and Tables

**Table 1:** MS/MS diagnostic mass spectra data for FAHFA molecular species detected in moose and caribou meats and antlers.

RT	Lipid Molecules	Molecular Species	Precursor *m*/*z*	Peak 1 *m*/*z*	Peak 2 *m*/*z*	Peak 3 *m*/*z*	Peak 4 *m*/*z*	Peak 5 *m*/*z*
21.66	FAHFA(32:0)	16:0-(9-O-16:0)	509.4575	255.2330	271.2279	253.2173	127.1128	127.1128
22.09	FAHFA(33:0)	15:0-(12-O-18:0)	523.4732	241.2173	299.2592	281.2486	169.1598	113.0972
22.53	FAHFA(34:0)	18:0-(9-O-16:0)	537.4888	283.2643	271.2279	253.2173	127.1128	127.1128
14.14	FAHFA(36:1)	18:1-(12-O-18:0)	563.5045	281.2486	299.2592	281.2486	169.1598	113.0972
11.81	FAHFA(36:3)	18:2-(9-O-18:1)	559.4732	279.2330	297.2435	279.233	127.1128	153.1285
13.70	FAHFA(38:3)	20:3-(5-O-18:0)	587.5045	305.2486	299.2592	281.2486	71.0502	211.2067
11.07	FAHFA(38:5)	20:4-(5-O-18:1)	583.4732	303.2330	297.2435	279.233	71.0502	209.1911
12.74	FAHFA(40:5)	20:3-(5-O-20:2)	611.5045	305.2486	323.2592	305.2486	71.0502	235.2067
11.62	FAHFA(40:6)	22:5-(5-O-18:1)	609.4888	329.2486	297.2435	279.233	71.0502	209.1911
11.05	FAHFA(40:7)	20:4-(5-O-20:3)	607.4732	303.2330	321.2435	303.2330	71.0502	233.1911
13.51	FAHFA(42:6)	20:3-(5-O-22:3)	637.5201	305.2486	349.2748	331.2643	71.0502	261.2224
11.16	FAHFA(42:8)	20:4-(5-O-22:4)	633.4888	303.2330	347.2592	329.2486	71.0502	259.2067
10.28	FAHFA(42:9)	20:4-(5-O-22:5)	631.4732	303.2330	345.2435	327.2330	71.0502	257.1911
11.56	FAHFA(44:9)	22:5-(5-O-22:4)	659.5045	329.2486	347.2592	329.2486	71.0502	259.2067

RT = retention time, Peak 1 = [FA − H]^−^, Peak 2 = [HFA − H]^−^, Peak 3 = [HFA − H − H_2_O]^−^; Peak 4 = [HFA fraction 1] and Peak 5 = [HFA fraction 2] show the position of hydroxy group on HFA backbone. Analysis conducted on an orbitrap accurate mass spectrometer operation at a resolution of 70,000 *m*/*z* in full scan mode and 35,000 *m*/*z* in MS/MS mode. Chromatography done using a high resolution Accucore C30 reverse phase column and a Dionex 3000 UHPLC system (See methods for further details).

**Table 2:** MS/MS diagnostic mass spectra for MAcDG molecular species detected in moose and caribou meats and antlers.

RT	Lipid Molecules	Molecular Species	Precursor *m*/*z*	Peak 1 *m*/*z*	Peak 2 *m*/*z*	Peak 3 *m*/*z*	Peak 4 *m*/*z*	Peak 5 *m*/*z*
22.50	MAcDG(34:4)	14:2/18:2/2:0	620.49	543.45	379.29	323.22	207.17	263.24
21.42	MAcDG(36:2)	16:0/18:2/2:0	652.55	575.51	379.29	355.29	239.24	263.24
22.10	MAcDG(36:1)	16:0/18:1/2:0	654.57	577.52	381.30	355.29	239.24	265.25
22.69	MAcDG(38:1)	18:0/18:1/2:0	682.60	605.56	381.30	383.32	267.27	265.25

RT = retention time, FA1 = Fatty acid at *sn-1*, FA2 = Fatty acid at *sn-2*, Precursor ion [M + NH_4_]^+^, Peak 1 [M − AcOH − NH_3_]^+^, Peak 2 [M − FA1 − NH_3_]^+^, Peak 3 [M − FA2 − NH_3_]^+^; Peak 4 [FA1 − H_2_O]^+^ and Peak 5 [FA2 − H_2_O]^+^ ketene ions.

## Abbreviations

C30RPLC	C30 reverse phase liquid chromatography
FAHFA	fatty acid esters of hydroxy fatty acid
DG	diglyceride
MAcDG	monoacetyldiglyceride
TG	triglyceride or commonly known as triacylglycerol
SCTG	short-chain triglyceride
MCTG	medium-chain triglyceride
PAHSA	palmitoyl hydroxy stearic acids
POHSA	palmitoleyl hydroxy stearic acids
OAHSA	oleoyl hydroxy stearic acids
ARA-5-HERA	arachidonyl 5-hydroxyeicosatrienoic acid
DHA-13-HLA	docosahexaenoyl 13-hydroxylinoleic acid
SFA	saturated fatty acid
MUFA	monounsaturated fatty acid
PUFA	polyunsaturated fatty acid
CM	Caribou Meat
MM-M	Moose Meat (male)
MM-F	Moose meat (female)
MA-T	Moose Antler (tips)
MA-B	Moose Antler (bases)
